# High FHL2 mRNA expression and its prognostic value in lung cancer

**DOI:** 10.18632/aging.204328

**Published:** 2022-10-10

**Authors:** Yan Jiao, Junyuan Wei, Zhibin Li, Jintao Zhou, Yunpeng Liu

**Affiliations:** 1 Department of Thoracic Surgery, The First Hospital of Jilin University, Changchun 130021, China; 2 Department of Hepatobiliary and Pancreatic Surgery, General Surgery Center, The First Hospital of Jilin University, Changchun 130021, China; 3 The Key Laboratory of Pathobiology, Ministry of Education, College of Basic Medical Sciences, Jilin University, Changchun 130021, China; 4 Department of Neurosurgery, The First Hospital of Jilin University, Changchun 130021, China

**Keywords:** FHL2, lung cancer, overall survival, relapse-free survival

## Abstract

Background: Lung cancer is the most frequent cancer globally with a high number of cancer-related deaths. The 4-and-a-half LIM domain protein 2 (FHL2) is an oncogenic gene, which promotes the proliferation, invasion, and metastasis of cancer cells. In this study, we aimed to demonstrate that lung cancer patients with high FHL2 expression have worse overall survival (OS) and relapse-free survival (RFS).

Methods: TCGA was used to study FHL2 mRNA expression. Nomograms were used to predict the relationship between FHL2 expression levels and survival. The qRT-PCR was used to detect the FHL2 expression in lung cancer cells. *In vitro* experiments including CCK-8 assay, wound healing, and Transwell assay were performed.

Results: This study comprised RNA-Seq gene expression data and clinical features for 1018 lung cancer patients. FHL2 was found to be overexpressed in lung cancer tissues. FHL2 demonstrated moderate diagnostic ability for lung cancer (AUC = 0.857). Kaplan–Meier curves and Cox regression analysis revealed the higher FHL2 expression with the poorer OS and RFS (*P* < 0.001). The nomogram results indicated that FHL2 could be used to predict the survival of lung cancer patients. GSEA analysis results show that high expression of FHL2 is related to glycolysis and unfolded protein reflection. FHL2 was highly expressed in lung cancer cells and related to their proliferation, migration, and invasion ability.

Conclusions: The high expression level of FHL2 in lung cancer can be used as an independent predictor of prognosis in clinical practice.

## INTRODUCTION

Lung cancer is the most frequent malignancy threatening human life with five-year survival rate of no more than 15% [1–3]. Since no specific clinical manifestations occur in the early stage of lung cancer, it is difficult to diagnose. Once there are apparent clinical symptoms, the malignancy has already developed into an advanced stage, when the prognosis of patients is extremely poor [4]. Spiral CT is often used for detection of early lung cancer. Early non-small cell lung cancer (NSCLC) patients are advised to receive surgery. Chemotherapy is usually administered in the perioperative period. High-dose stereotactic radiation therapy can be also used and usually sustained for 6 weeks. Molecular targeted therapy is often used for advanced lung cancer. For lung cancer, biomarkers play important roles in diagnosis, treatment and improving prognosis. However, many biomarkers are limited to specific molecular types of lung cancer, thus provoking searches for novel biomarkers to forecast prognosis on a grander scale.

FHL2, a multifunctional scaffolding protein, has been shown to modulate gene transcription and signaling cascades [5]. Previous reports have shown that FHL2 facilitates cell proliferation in glioblastoma [6], gastric, colon [7], and cervical [8] cancers. Specifically, FHL2 interacts with EGFR to promote glioblastoma growth [6]. FHL2 suppression inhibits gastric and colon carcinogenesis [7]. FHL2 facilitates MDM2-mediated degradation of IER3 to regulate proliferation of cervical cancer [8]. FHL2 also shows a great potential to diagnose malignancies, become a therapeutic target and predict prognosis of cancer. Nevertheless, the diagnostic and prognostic value of FHL2 in lung cancer is still unclear.

Herein, we assessed the correlation between FHL2 expression in lung cancer and clinicopathologic characteristics through analyzing data from The Cancer Genome Atlas (TCGA) database. The receiver operating characteristic (ROC) curves were plotted to analyze the diagnostic value of FHL2. We further evaluated the independent prognostic ability of FHL2 expression for relapse-free survival (RFS) and overall survival (OS) through the Kaplan–Meier curve, subgroup analysis, univariate Cox analysis and multivariate Cox analysis. Besides, the ability of FHL2 to predict RFS and OS was reflected by nomogram. Finally, the relationship of high FHL2 expression with glycolysis and unfolded protein reflection was explored by GSEA analysis.

## RESULTS

### Patient features

The RNA expression information and related clinical data were acquired from TCGA database. In total, 1018 patients with lung cancer, including 407 females and 611 males were analyzed. Moreover, 109 patients were <55 years old, and 881 patients were ≥55 years old. The detailed clinical features of the patients derived from TCGA were demonstrated in Table 1. There were significant differences (*P* < 0.05) in histological type, T, N classification, and vital status. However, no statistical differences were found in other parameters.

**Table 1:** Relationship between FHL2 expression and clinicopathological parameters in patients with lung cancer.

**Parameter**		***N***	**FHL2**				**X^2^**	***P***
			**High**	**%**	**Low**	**%**		
Age	<55	109	58	(11.76)	51	(10.26)	0.4276	0.5132
	≥55	881	435	(88.24)	446	(89.74)		
Gender	Female	407	204	(40.48)	203	(39.49)	0.0654	0.7981
	Male	611	300	(59.52)	311	(60.51)		
Histological type	lung adenocarcinoma	517	284	(56.35)	233	(45.24)	12.1306	0.0005^***^
	lung squamous cell carcinoma	502	220	(43.65)	282	(54.76)		
Stage	I	521	249	(49.9)	272	(53.65)	3.8310	0.2810
	II	284	138	(27.66)	146	(28.8)		
	III	168	94	(18.84)	74	(14.6)		
	IV	33	18	(3.61)	15	(2.96)		
T classification	T1	284	115	(22.82)	169	(32.88)	13.9650	0.0048^**^
	T2	571	303	(60.12)	268	(52.14)		
	T3	118	65	(12.9)	53	(10.31)		
	T4	42	20	(3.97)	22	(4.28)		
	TX	3	1	(0.2)	2	(0.39)		
N classification	N0	652	321	(63.69)	331	(64.52)	14.1067	0.0057^**^
	N1	227	99	(19.64)	128	(24.95)		
	N2	114	73	(14.48)	41	(7.99)		
	N3	7	2	(0.4)	5	(0.97)		
	NX	17	9	(1.79)	8	(1.56)		
M classification	M0	758	374	(74.8)	384	(75.29)	0.6057	0.7575
	M1	32	18	(3.6)	14	(2.75)		
	MX	220	108	(21.6)	112	(21.96)		
Radiation therapy	NO	782	387	(88.15)	395	(87.2)	0.1111	0.7388
	YES	110	52	(11.85)	58	(12.8)		
Residual tumor	R0	743	358	(90.18)	385	(90.16)	0.7633	0.8763
	R1	25	12	(3.02)	13	(3.04)		
	R2	8	5	(1.26)	3	(0.7)		
	RX	48	22	(5.54)	26	(6.09)		
Vital status	Deceased	404	218	(43.25)	186	(36.19)	5.0188	0.0251^*^
	Living	614	286	(56.75)	328	(63.81)		
Sample type	Primary Tumor	1017	502	(99.6)	515	(100)	0.5229	0.4696
	Recurrent Tumor	2	2	(0.4)	0	(0)		

X represents unknown, ^*^P < 0.05; ^**^P < 0.01; ^***^P < 0.001.

### High FHL2 mRNA expression in lung cancer

**Figure 1:**
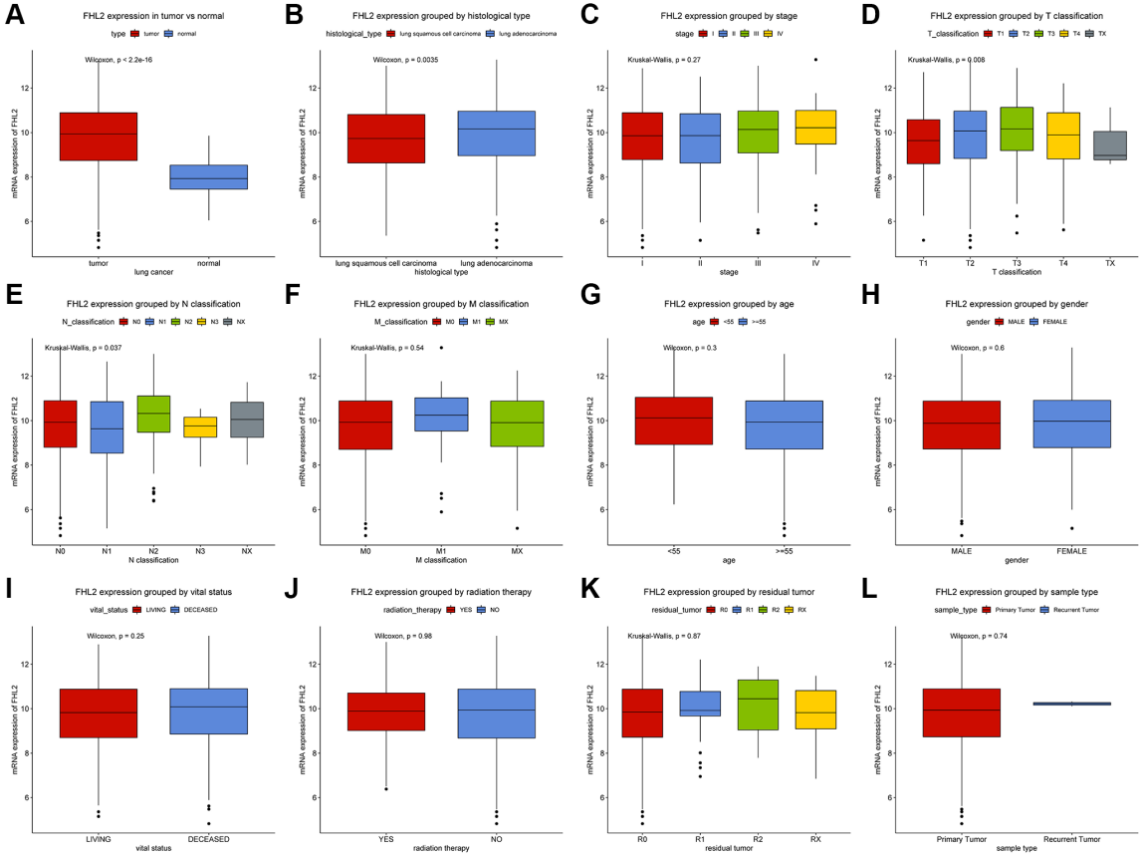
**Expression of FHL2 in normal population and lung cancer patients and its relationship with clinicopathological parameters.** (**A**) The expression difference of FHL2 between normal population and lung cancer patients. (**B**, **C**) Expression of FHL2 in different histological types and stages. (**D**–**F**) The relationship between FHL2 expression and T classification, N classification, and M classification. (**G**–**L**) The relationship between FHL2 expression and age, gender, vital status, radiotherapy, residual tumor, and recurrent tumors. ^*^P < 0.05; ^**^P < 0.01.

The mRNA expression of FHL2 in lung tumor tissue was significantly higher (*P* < 2.2e-16) than that in normal lung tissue (Figure 1A). Moreover, different FHL2 expression levels were observed (Figure 1B–1L). Lung adenocarcinoma patients had higher FHL2 expression levels (*P* < 0.01) than lung squamous cell carcinoma patients (Figure 1B). Despite the *P* value was bigger than 0.05, the result indicated a gradual increase with higher stages (Figure 1C). FHL2 mRNA expression levels of T, N, M classifications are illustrated in Figure 1D–1F. Despite statistical differences are not significant, we still identified patients who were female, <55 years old, deceased and treated with radiation therapy had higher FHL2 expression levels than patients who were male, ≥55 years old, living, and not treated with radiation therapy respectively (Figure 1G–1J). With regard to residual tumor, 4 groups had similar FHL2 expression, while R2 had the higher FHL2 expression level compared to the other groups (Figure 1K). Patients with primary tumor seemed to have lower FHL2 expression levels than patients with recurrent tumor (Figure 1L, *P* = 0.74), but further studies need to be conducted due to only 2 patients with recurrent tumor.

### Capability of FHL2 to diagnose lung cancer

**Figure 2:**
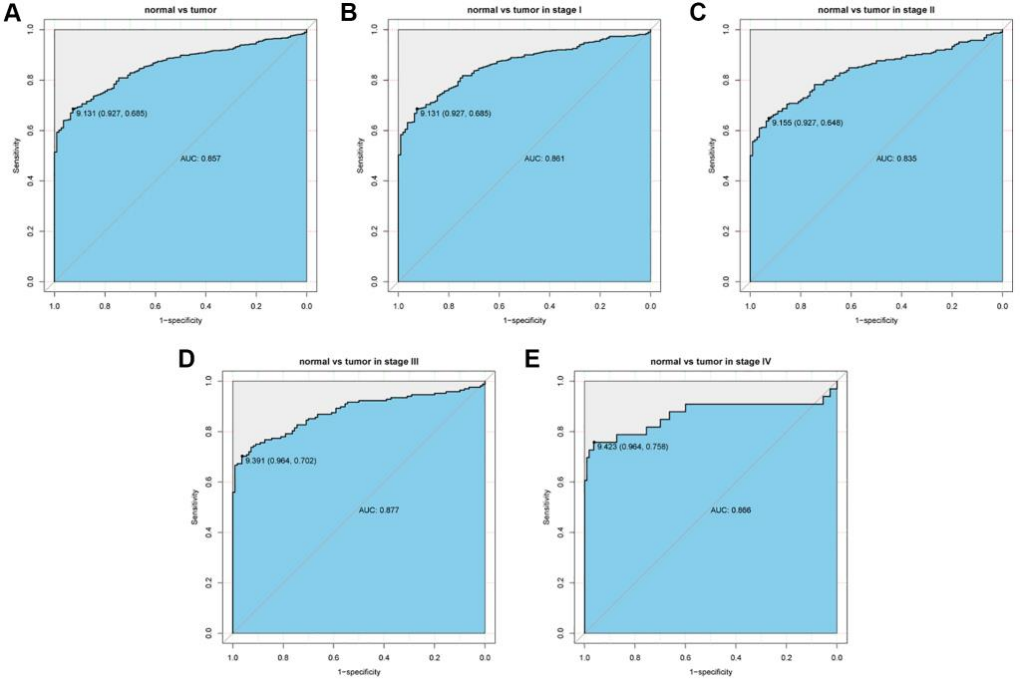
**ROC curve to assess the diagnostic ability of FHL2 at different stages.** (**A**) The diagnostic value of FHL2 in lung cancer patients. (**B**–**E**) Diagnostic value of FHL2 in patients with stage I, stage II, stage III, and stage IV.

The ROC curves were utilized to analyze the diagnostic value of FHL2. A moderate diagnostic capability in lung cancer was observed with the AUC of 0.857 (Figure 2A). We also evaluated the diagnostic ability of FHL2 in the different stages. The similar results were found (Figure 2B–2E) that AUC values were 0.861 (stage I), 0.835 (stage II), 0.877 (stage III) and 0.866 (stage IV), respectively.

### Relationships between clinical features and FHL2 expression

The patients were divided into the low- and high- groups according to the threshold of FHL2 level determined from the ROC curve. As demonstrated in Table 1, high FHL2 mRNA expression was highly correlated with histological type (*P* = 0.0005), T classification (*P* = 0.0048), N classification (*P* = 0.0057) and vital status (*P* = 0.0251) in the light of Chi-square tests.

### FHL2 mRNA expression is associated with overall survival

**Figure 3:**
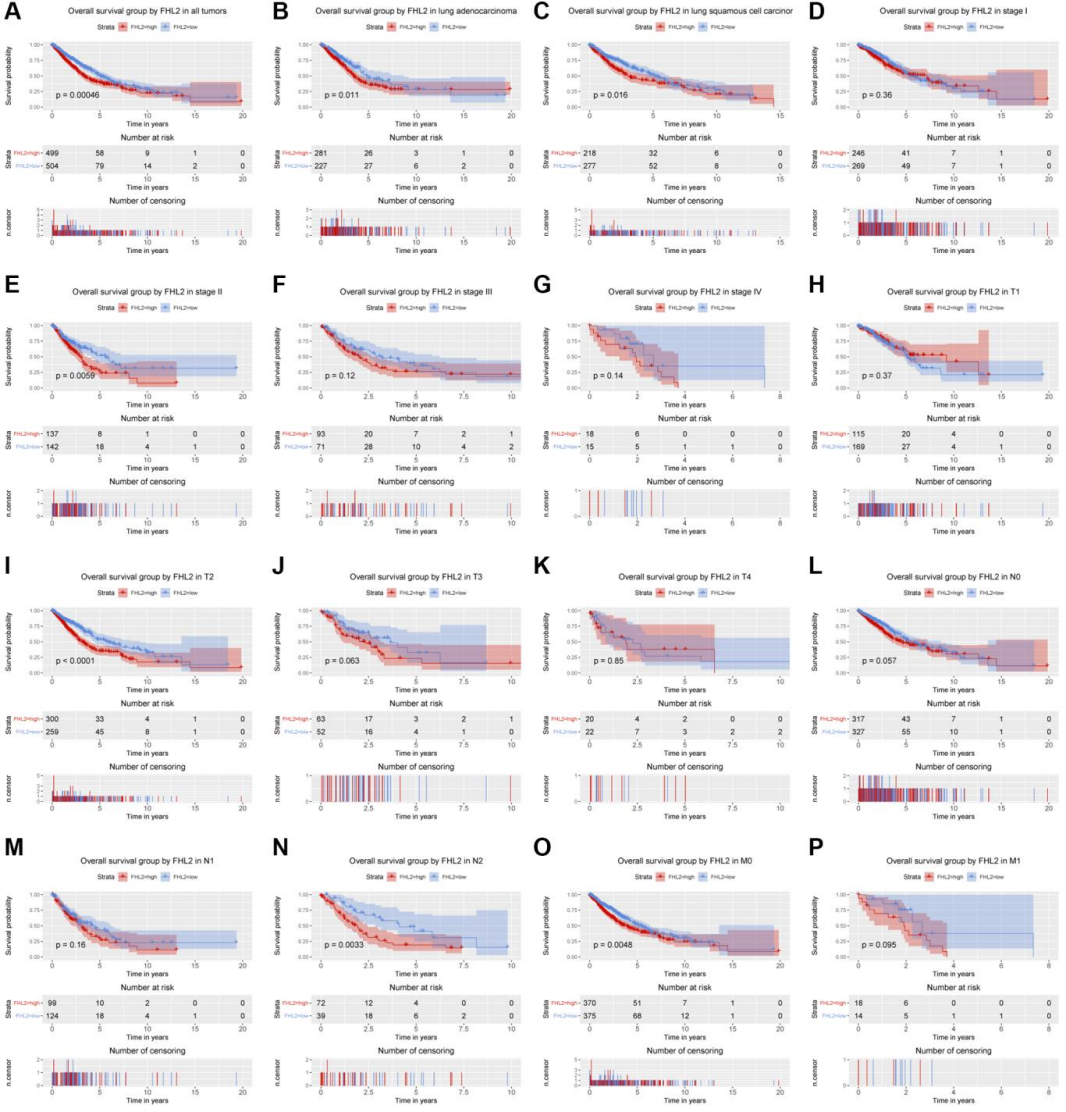
**The relationship between FHL2 mRNA expression and overall survival.** (**A**) Overall survival group by FHL2 in all tumors. (**B**–**P**) Overall survival group by FHL2 in lung adenocarcinoma, lung squamous cell carcinoma, stage I, stage II, stage III, stage IV, T1, T2, T3, T4, N0, N1, N2, M0, M1.

As demonstrated in Figure 3, the Kaplan–Meier curve with log rank tests showed the relationship between FHL2 mRNA expression and overall survival. High FHL2 mRNA expression was remarkably related with poor overall survival (Figure 3A, *P* = 0.0005). The further subgroup analysis (Figure 3B–3P) revealed that high FHL2 expression was associated with poor OS of patients with lung adenocarcinoma (*P* = 0.0110), lung squamous cell carcinoma (*P* = 0.0160), stage II (*P* = 0.0059), T2 (*P* < 0.0001), N2 (*P* = 0.0033) and M0 (*P* = 0.0048). Additionally, although *P* value was bigger than 0.05, we still identified that high FHL2 expression was correlated with poor OS patients with stage I, stage III, stage IV, T1, T3, T4, N0, N1 and M1 NSCLC.

**Figure 4:**
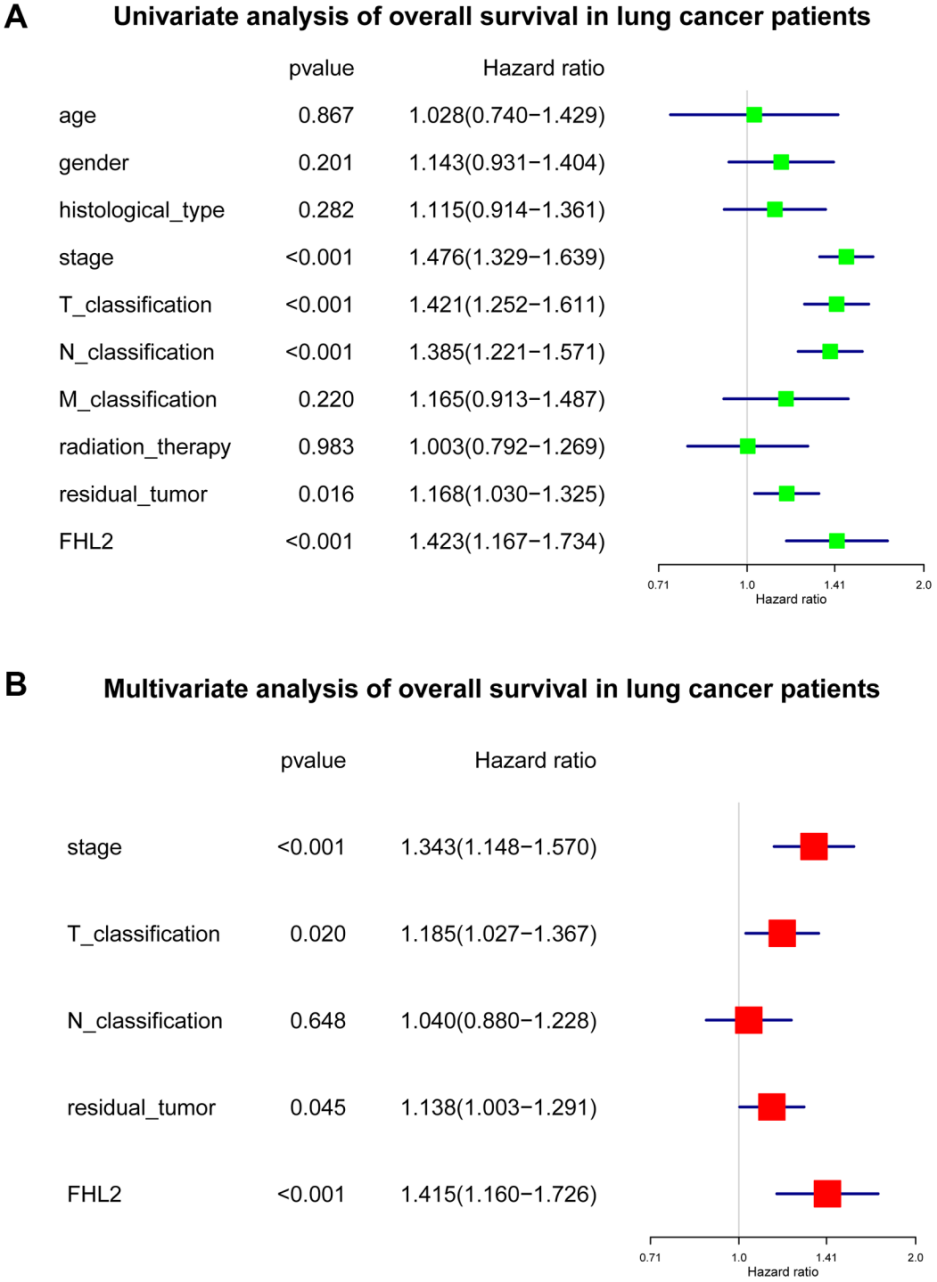
**The subsequent multivariate analysis about FHL2 and overall survival in lung cancer.** (**A**) Univariate analysis of overall survival in lung cancer patients. (**B**) Multivariate analysis of overall survival in lung cancer patients. ^*^P < 0.05; ^**^P < 0.01; ^***^P < 0.001.

Univariate Cox analysis identified several crucial variables, and the subsequent multivariate analysis confirmed that stage [hazard ratio (HR): 1.343, 95% confidence interval (CI): 1.148–1.570, *P* < 0.001], T classification (HR: 1.185, 95% CI: 1.027–1.367, *P* = 0.020), residual tumor (HR: 1.138, 95% CI: 1.003–1.291, *P* = 0.045) and FHL2 expression (HR: 1.415, 95% CI: 1.160–1.726, *P* < 0.001) had independent prognostic value for OS of patients with lung cancer. (Figure 4).

### FHL2 mRNA expression is correlated with relapse-free survival

**Figure 5:**
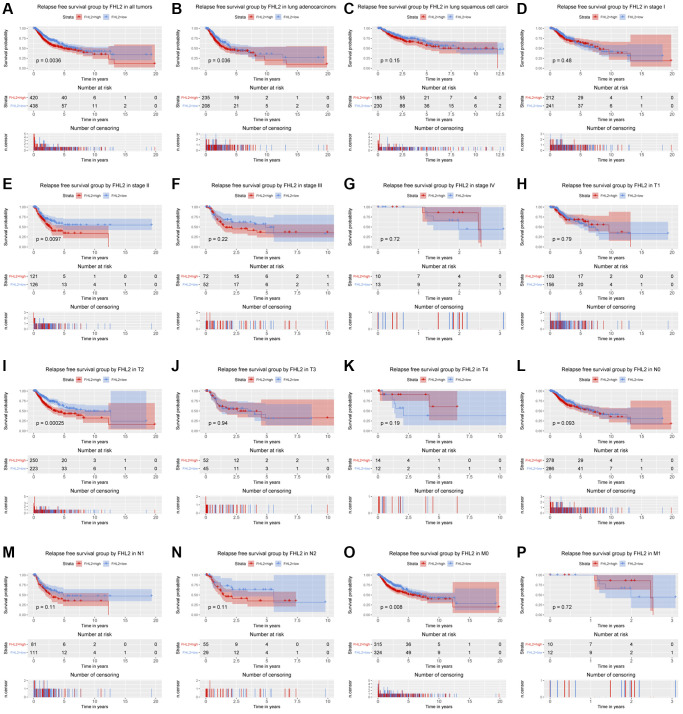
**The relationship between FHL2 mRNA expression and relapse free survival.** (**A**) Relapse free survival group by FHL2 in all tumors. (**B**–**P**) Relapse free survival group by FHL2 in lung adenocarcinoma, lung squamous cell carcinoma, stage I, stage II, stage III, stage IV, T1, T2, T3, T4, N0, N1, N2, M0, M1.

**Figure 6:**
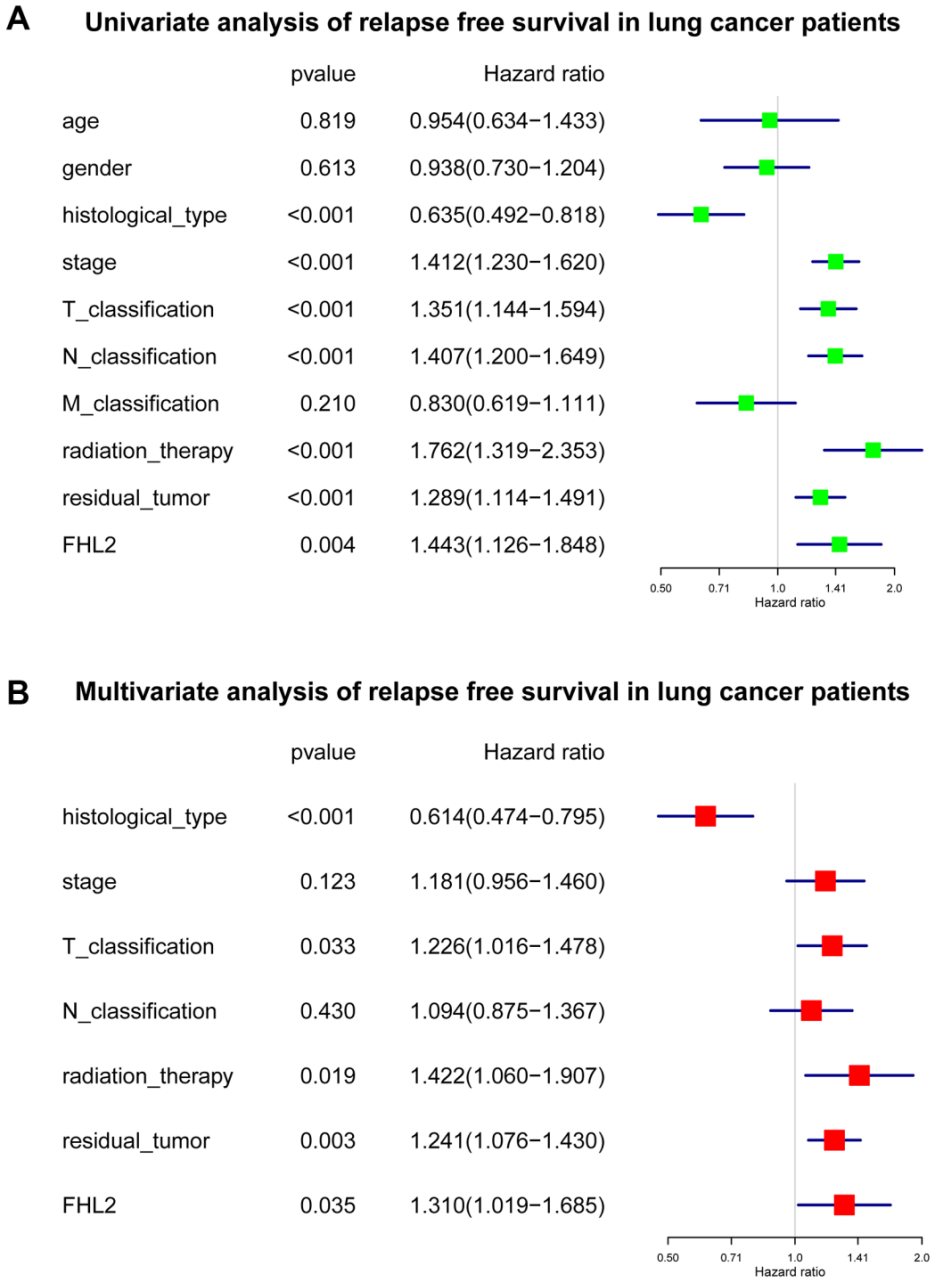
**The subsequent multivariate analysis about FHL2 and relapse free survival in lung cancer.** (**A**) Univariate analysis of relapse free survival in lung cancer patients. (**B**) Multivariate analysis of relapse free survival in lung cancer patients. ^*^P < 0.05; ^**^P < 0.01; ^***^P < 0.001.

The Kaplan–Meier survival curve was utilized to evaluate the relationship between FHL2 expression and RFS (Figure 5). Similar to the outcomes above, high FHL2 expression demonstrated a close association with lung adenocarcinoma, stage II, T2, and M0 lung carcinoma. High FHL2 expression showed remarkable prognostic value (*P* = 0.0036). More importantly, we plotted ROC curves to assess the value of FHL2 in predicting one-, three-, and even five-year RFS in lung cancer patients. Additionally, univariate Cox analysis was utilized to select the crucial prognostic factors. Furthermore, multivariable analysis was implemented to modify interaction between factors. Eventually, FHL2 expression was validated as an independent prognostic factor of patients with NSCLC (Figure 6).

### FHL2 can serve as an independent predictor of survival

To evaluate the value of FHL2 as an independent indicator for predicting patient survival, we used the data downloaded from the TCGA database to draw a nomogram of FHL2 predicting patient survival and progression-free survival, and reflected the prediction results through the ROC curve.

**Figure 7:**
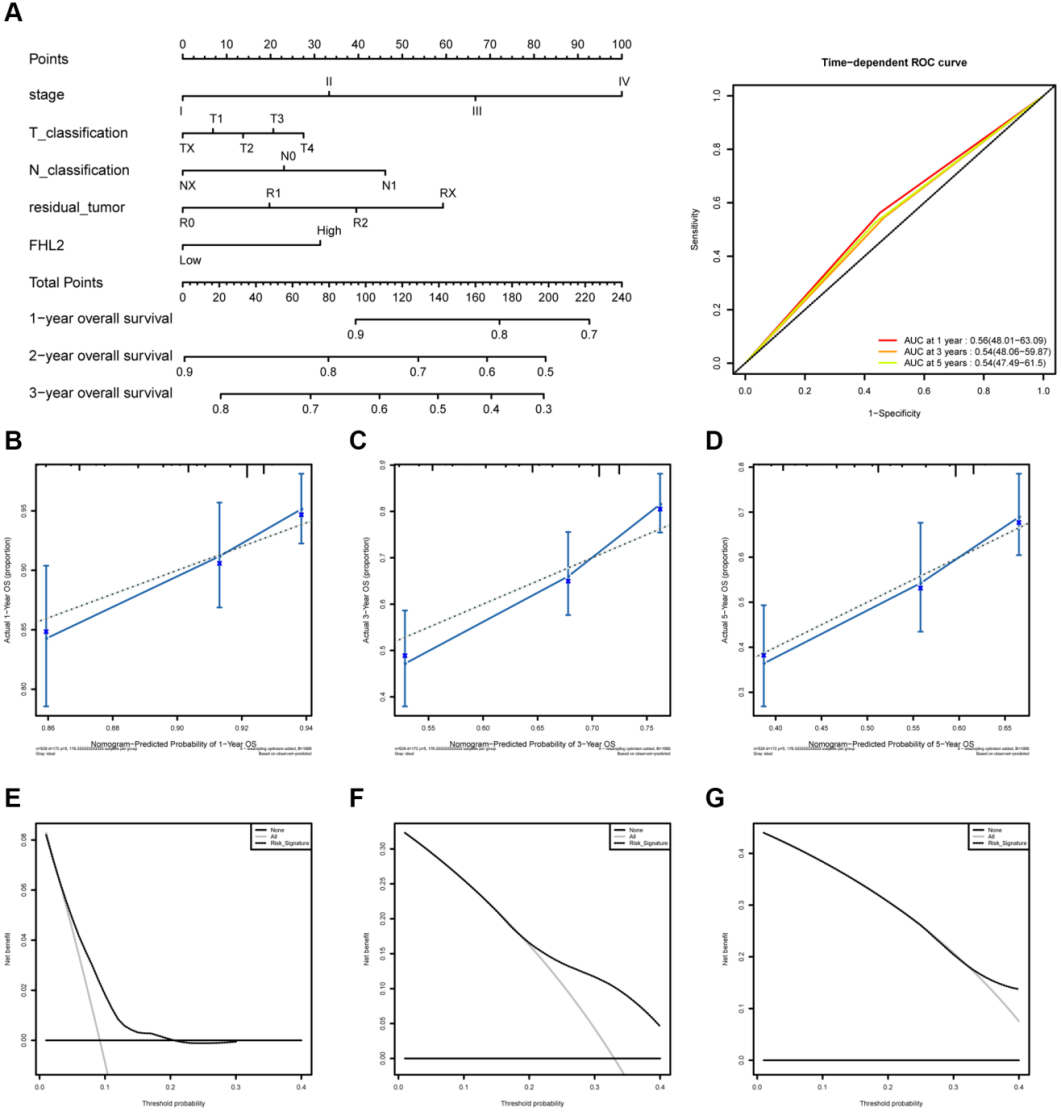
**The ROC curves and nomogram about FHL2 and overall survival in lung cancer.** (**A**) ROC curves evaluating the value of FHL2 for predicting overall survival in lung cancer patients. (**B**) Nomogram predicted 1-year overall survival versus actual 1-year overall survival. (**C**) Nomogram predicted 3-year overall survival versus actual 3-year overall survival. (**D**) Nomogram predicted 5-year overall survival versus actual 5-year overall survival. (**E**–**G**) Decision curve analysis reflects the feasibility of FHL2 in predicting 1-year, 3-year, and 5-year overall survival of patients.

As shown in Figure 7, the results showed that patients with high FHL2 expression had shorter OS. Besides, the patients with higher stages, higher T classification, higher N classification, or higher residual tumor also had shorter overall survival. The time-dependent ROC curve exhibited a moderate diagnostic capability with AUC of 0.56 at 1 year, 0.54 at 3 and 5 years (Figure 7A). The nomogram-predicted probability of 1-year, 3-year, and 5-year OS were close to the corresponding actual OS (Figure 7B–7D). At the same time, the decision curve was used to reflect the FHL2 prediction model, and the results showed that patients with high FHL2 expression could predict shorter OS (Figure 7E–7G).

**Figure 8:**
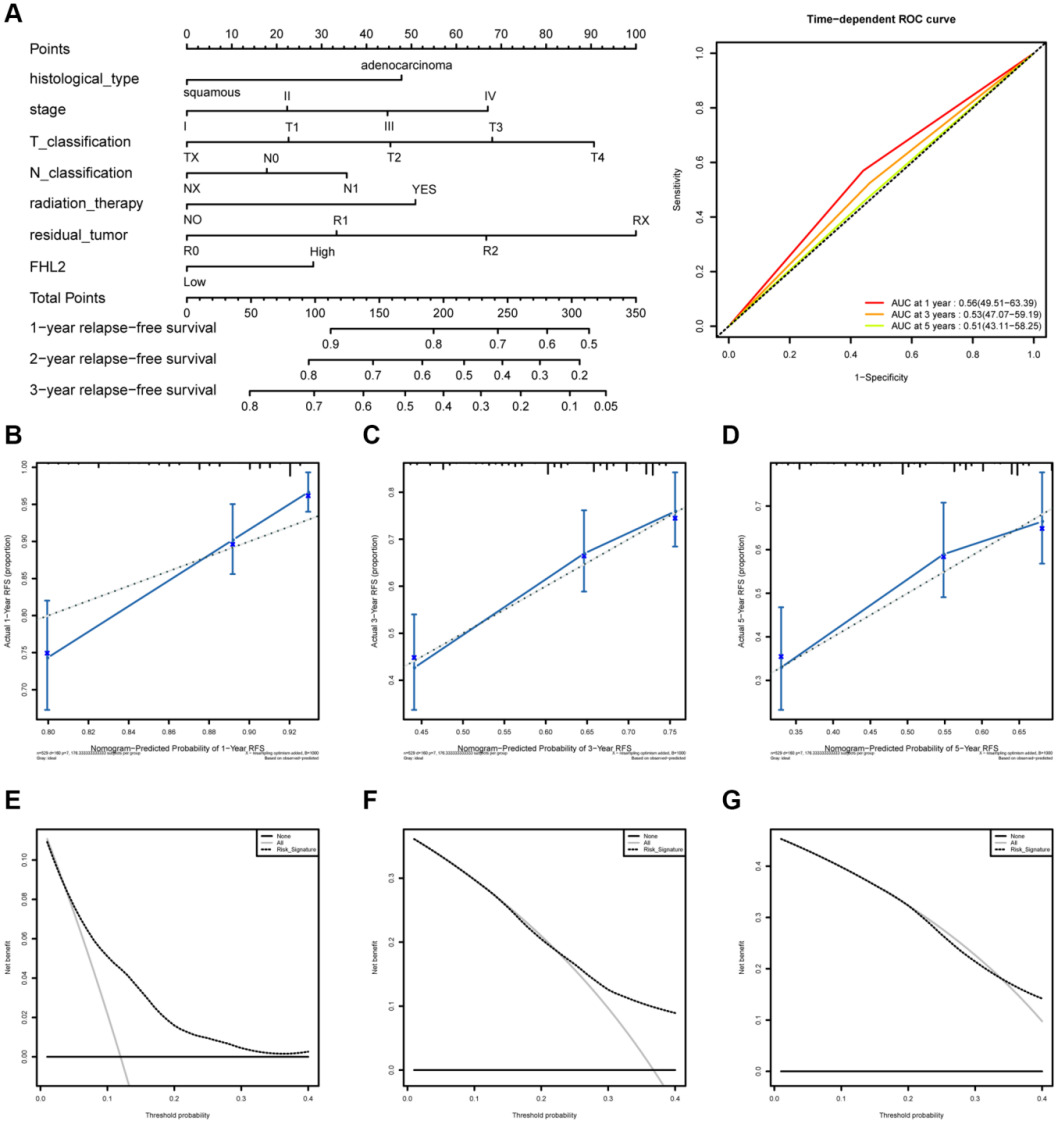
**The ROC curves and nomogram about FHL2 and RFS in lung cancer.** (**A**) ROC curves evaluating the value of FHL2 for predicting RFS in lung cancer patients. (**B**) Nomogram predicted 1-year RFS versus actual 1-year RFS. (**C**) Nomogram predicted 3-year RFS versus actual 3-year RFS. (**D**) Nomogram predicted 5-year RFS versus actual 5-year RFS. (**E**–**G**) Decision curve analysis reflects the feasibility of FHL2 in predicting 1-year, 3-year, and 5-year RFS of patients.

As shown in Figure 8, the results showed that patients with high FHL2 expression had shorter RFS. Similarly, the patients with adenocarcinoma, higher stages, higher T classification, or higher N classification also had shorter RFS. The time-dependent ROC curve exhibited a moderate diagnostic capability with AUC of 0.56 at 1 year, 0.53 at 3 years, and 0.51 at 5 years (Figure 8A). The nomogram-predicted probability of 1-year, 3-year, and 5-year RFS were close to the corresponding actual RFS (Figure 8B–8D). At the same time, the decision curve was used to reflect the FHL2 prediction model, and the results showed that patients with high FHL2 expression could predict shorter RFS (Figure 8E–8G).

### Patients with high FHL2 expression are more prone to glycolysis and unfold protein response

To further explore the mechanism by which FHL2 affects lung cancer progression, the GSEA analysis through the database obtained from TCGA was further performed to examine the correlation between high FHL2 expression and Glycolytic and unfolded protein responses. The relevant data scores are shown in Table 2.

**Table 2:** Gene set for FHL2 high group.

**Name**	**Size**	**ES**	**NES**	**NOM *p*-val**	**FDR *q*-val**
HALLMARK_GLYCOLYSIS	199	0.549744	2.132682	0	0.019725
HALLMARK_UNFOLDED_PROTEIN_RESPONSE	113	0.477332	2.022087	0.002028	0.022602

^*^P < 0.05; ^**^P < 0.01.

**Figure 9:**
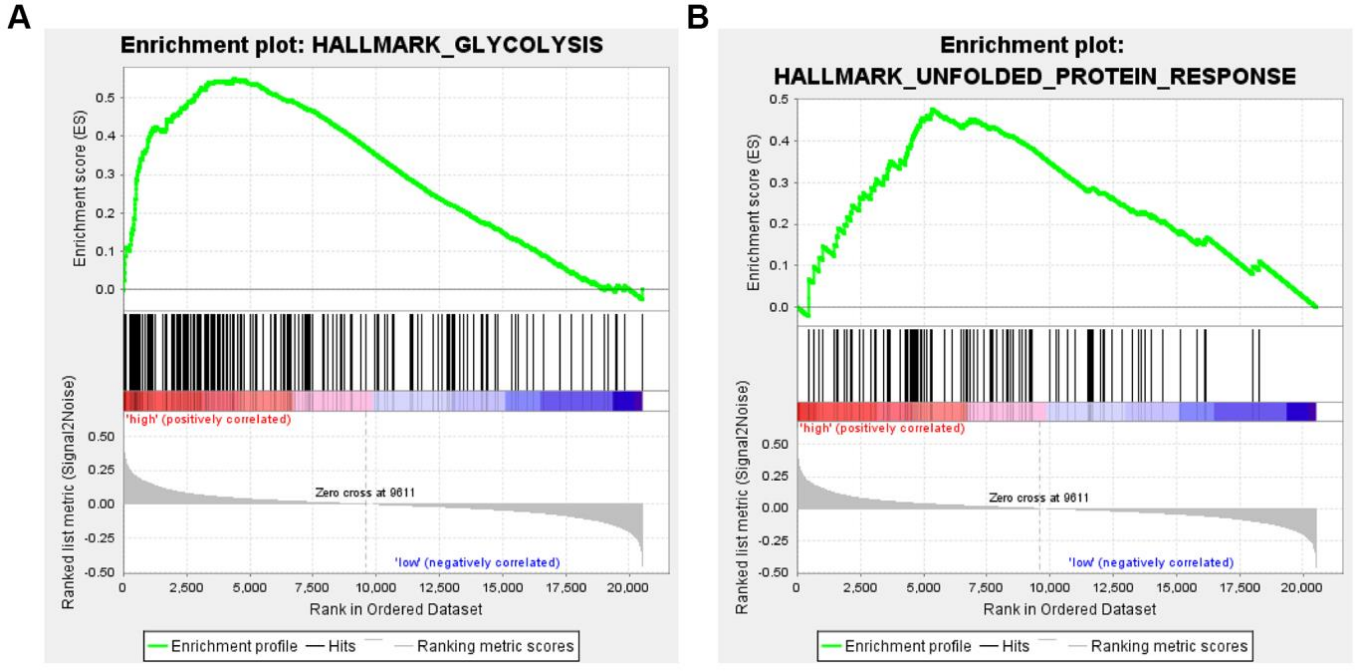
**Enrichment analysis of FHL2 expression and glycolytic and unfolded protein responses.** (**A**) High expression of FHL2 is enriched in the Glycolytic pathway. (**B**) High FHL2 expression is highly correlated with unfolded protein response.

The results showed that high FHL2 expression was highly correlated with Glycolysis and unfolded protein responses (Figure 9). This suggests that FHL2 may affect lung cancer progression by regulating Glycolysis and inducing unfolded protein response.

### FHL2 knockdown inhibits proliferation, migration, and invasion of NSCLC cells

**Figure 10:**
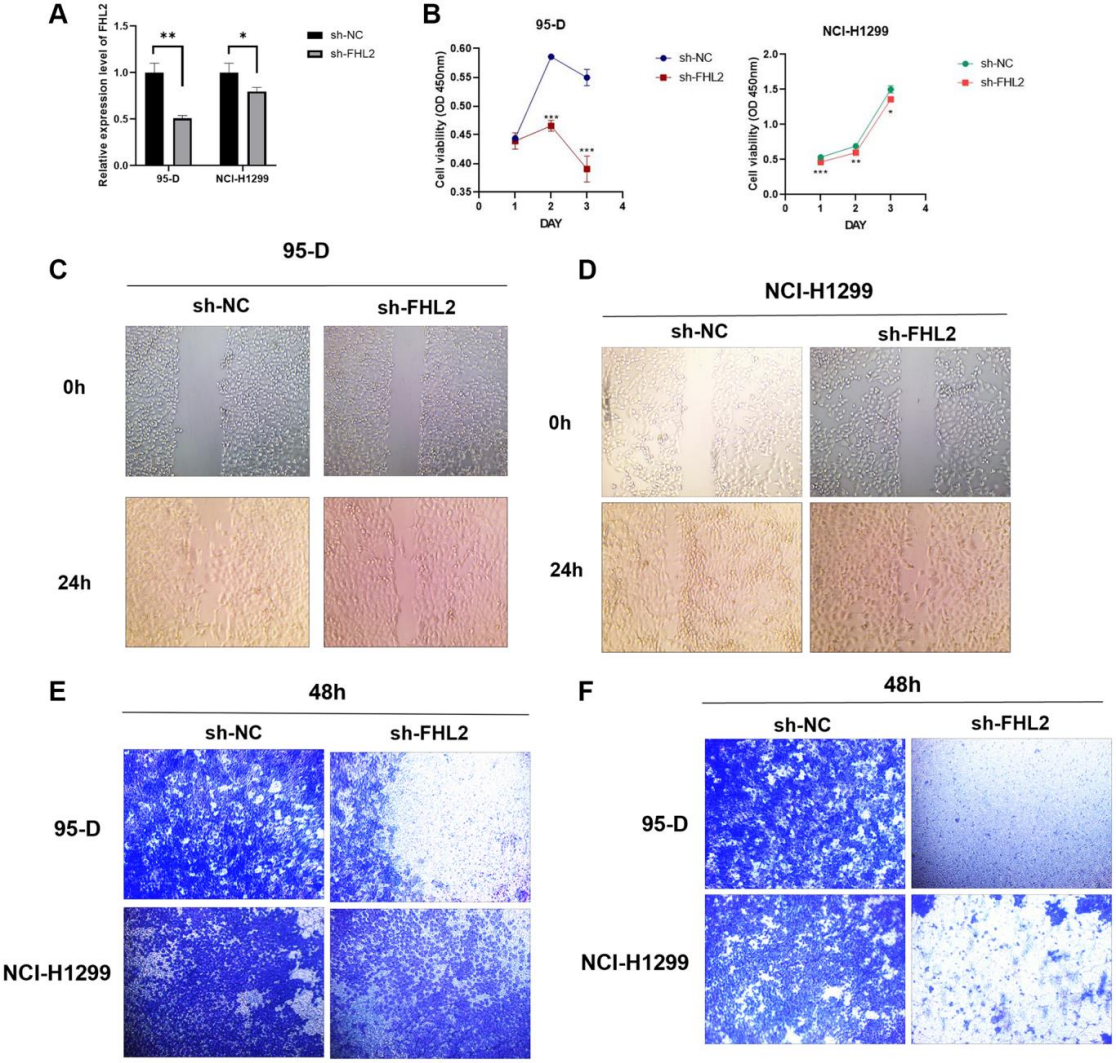
**Effects of FHL2 on proliferation, migration, and invasion of NSCLC.** (**A**) qRT-PCR detected the efficiency of transfection. (**B**) CCK8 detected the viability of NSCLC cells. (**C**, **D**) Wound healing experiment detected the migration ability of NSCLC cells. (**E**) Transwell experiment detected the migration ability of NSCLC cells. (**F**) Transwell experiment detected the invasive ability of NSCLC cell. ^*^P < 0.05; ^**^P < 0.01; ^***^P < 0.001.

In order to explore the effect of FHL2 on the biological behavior of NSCLC *in vitro*, we selected 95-D and NCI-H1299 cell lines with high invasiveness and active proliferation for subsequent experiments. We established A549 and NCI-H1299 cells stably expressing FHL2 targeted shRNA, and detected the transfection efficiency by qRT-PCR (Figure 10A). The results of CCK8 assays showed that the proliferation ability of the cells was markedly decreased in sh-FHL2 group compared to sh-NC group (Figure 10B). This indicates that the proliferation ability of cells was inhibited after silencing FHL2. We further explored the effects of FHL2 on NSCLC cell migration and invasion. As shown in Figure 10C–10F, the number of migrated and invaded cells were decreased in sh-FHL2 group compared to sh-NC group. These experiments proved that FHL2 may play an important role in promoting NSCLC by promoting the migration, invasion, and proliferation ability of NSCLC cells. This finding provides a new idea for the clinical treatment of NSCLC.

## DISCUSSION

Globally, NSCLC is the second most frequent malignancy with the mortality gradually increased [2, 3]. New molecular biomarkers that can improve the survival and predicting prognosis of NSCLC are urgently required. This study focused on FHL2 mRNA expression and revealed the crucial role of FHL2 in NSCLC. In addition, FHL2 expression exhibited independent prognostic value of NSCLC, making it a potential biomarker in the near future.

FHL2 comprises four and one-half highly preserved cysteine-rich LIM homeodomains. These unique structures lead to interaction of FHL2 with many different proteins [9]. The function of FHL2 in cancer is especially intriguing because it may function as a tumor inhibitor or an oncogenic protein [5]. Previous reports have shown that FHL2 facilitates cell proliferation in glioblastoma [6], gastric, colon [7], and cervical [8] cancers, but inhibits neuroblastoma [10] and myeloid malignancies [11]. In this study, FHL2 has been observed to be overexpressed in patients with lung malignancy (*P* < 2.2e^−16^). Additionally, we also observe that higher FHL2 was corelated with poor outcomes of NSCLC patient.

The detailed molecular mechanisms of the oncogenic effects of FHL2 remain poorly understood. FHL2 has been involved in the Wnt/β-catenin signaling cascades [9]. Wingless/integrated-mediated signal pathways are known to adjust stemness and development, but are also highly associated with cancer [12–14]. Activated Wnt signal pathway has been identified to mediate NSCLC initiation and progression by modulating cell proliferation, apoptosis, and epithelial-to-mesenchymal transition [15, 16].

The Wnt signal pathway is commonly separated from β-catenin-dependent and β-catenin-independent signaling [12, 13]. Wnt ligand combining to Frizzled receptors and LRP co-receptors activates the β-catenin-dependent pathway. These interactions interrupt the APC/Axin/ CK1A/GSK3β complex, which lead to β-catenin translocating and amassing in the nucleus [12, 13]. FHL2 was first reported to be a coactivator of β-catenin [17] that drove differentiation of mouse myoblasts [18]. In colon cancer and osteosarcoma, FHL2 can make nuclear β-catenin stable, prompting β-catenin transactivation activity [19, 20]. FHL2 was also reported to promote tubular EMT through modulating β-catenin signaling pathway in fibrotic kidneys [21]. In agreement with aberrant Wnt/β-catenin signaling, the gene expression of FHL2 has been shown to be dysregulated in a wide variety of cancers [8, 20, 22, 23]. As a coactivator of β-catenin [17], FHL2 activates the Wnt signaling cascade, EMT signals, and downstream cell cycle progression.

Consistently, FHL2 was highly expressed in lung cancer patients. At the same time, the results of the boxplot indicated that the high expression of FHL2 was associated with the poor clinical characteristics of the patients. We found that high FHL2 expression was associated with worse OS and RFS. Also, through nomogram and decision curve results, we found that FHL2 can serve as an independent predictive model for predicting patient OS and RFS.

GSEA analysis revealed findings on the mechanism by which FHL2 affects lung cancer progression. We found that FHL2 contributes to lung cancer progression by inducing glycolysis and promoting unfolded protein responses. However, how FHL2 regulates glycolysis and unfolded protein reflection remains to be further explored. In addition, *in vitro* experiments also showed that FHL2 is highly expressed in lung cancer cells, and the high expression of FHL2 is related to the enhancement of the proliferation, migration, and invasion ability.

In conclusion, FHL2 has great potential to predict the prognosis of lung cancer. The further study needs to be implemented to explore the molecular mechanism. *In vivo* study may further confirm these discoveries. The high expression level of FHL2 in lung cancer can be used as an independent predictor of prognosis in clinical practice.

## MATERIALS AND METHODS

### Clinical data

The complete RNA-Seq expression information and corresponding clinical characteristics were acquired from TCGA database. The FHL2 mRNA expression was calculated as log_2_ (x + 1) and converted into RSEM normalized counts.

### Nomogram plotting

The lung cancer patients were grouped according to FHL2 expression. Based on the different FHL2 expression, the T/N/M classification, stage, grade, and prognosis of patients were compared.

### GSEA assay

The TCGA database was screened, followed by performing GSEA analysis through the online website to explore the relationship between FHL2 expression and Glycolysis and unfolded protein reflection.

### Cell culture

Human lung cancer cell lines 95-D and NCI-H1299 were cultured in 1640 medium containing 10% fetal bovine serum and 1% penicillin-streptomycin solution in a 5% CO_2_ humidified incubator at 37°C.

### Plasmid and transfection

The sh-FHL2 plasmid and sh-control plasmid were purchased (Miaoling Bio, China), followed by transfection into the cell and generation of the stable clone.

### RT-qPCR

All the reagents and kits were purchased from Invitrogen (USA). The TRIzol was used to extract total RNA. The reverse transcription was performed using 1 mL of extracted RNA. Finally, the real time quantitative PCR (RT-qPCR) was carried out.

### Wound healing assay

The cells were scratched to form a 1-mm wide gap, and cultured for 2 days [24]. In order to measure the breadth of the injured region, pictures were obtained at 0 and 48 hours.

### Transwell invasion assay

The plates with 8 μm pore were used, covering with Matrigel (Corning, USA). In the Transwell’s upper chamber, cells were added. Then, the crystal violet-stained cells invasive to the lower surface were observed.

### CCK-8 assay

The cells were seeded with density of 1 × 10^5^/well [25]. After culturing for 24 h, DOX was added. Then, 10 μL CCK-8 reagent was added and the absorbance at 450 nm was measured.

### Statistical analysis

All materials were retrospectively evaluated using R (version 3.5.1) [26]. We utilized non-parametric rank sum examinations to evaluate FHL2 mRNA expression levels. Wilcoxon rank sum examinations were utilized to contrast two subgroups, and Kruskal–Wallis tests were utilized for comparing multiple subgroups. We utilized the pROC package to depict Receiver Operating Characteristic (ROC) curves for the analysis of FHL2 diagnostic ability through calculating area under ROC curves (AUC) values and measuring ideal cutoff point [27]. In addition, association between clinical characteristics and FHL2 expression was evaluated by chi-square tests with Fisher’s exact test. Kaplan–Meier curve was utilized to study survival status. Univariate Cox and multivariate Cox analysis were utilized to study the independent prognostic ability of FHL2 in lung cancer was identified through. *P* < 0.05 was statistically significant.
